# Three-dimensional in vitro follicle growth: overview of culture models, biomaterials, design parameters and future directions

**DOI:** 10.1186/1477-7827-8-119

**Published:** 2010-10-14

**Authors:** Nina Desai, Anastasia Alex, Faten AbdelHafez, Anthony Calabro, James Goldfarb, Aaron Fleischman, Tommaso Falcone

**Affiliations:** 1 Cleveland Clinic Fertility Center, Department of OB/GYN and Women's Health Institute, Cleveland Clinic Foundation, Cleveland, Ohio, USA; 2 Department of Biomedical Engineering, Lerner Research Institute, Cleveland Clinic Foundation, Cleveland, Ohio, USA

## Abstract

In vitro ovarian follicle culture is a new frontier in assisted reproductive technology with tremendous potential, especially for fertility preservation. Folliculogenesis within the ovary is a complex process requiring interaction between somatic cell components and the oocyte. Conventional two-dimensional culture on tissue culture substrata impedes spherical growth and preservation of the spatial arrangements between oocyte and surrounding granulosa cells. Granulosa cell attachment and migration can leave the oocyte naked and unable to complete the maturation process. Recognition of the importance of spatial arrangements between cells has spurred research in to three-dimensional culture system. Such systems may be vital when dealing with human primordial follicles that may require as long as three months in culture. In the present work we review pertinent aspects of in vitro follicle maturation, with an emphasis on tissue-engineering solutions for maintaining the follicular unit during the culture interval. We focus primarily on presenting the various 3-dimensional culture systems that have been applied for in vitro maturation of follicle:oocyte complexes. We also try to present an overview of outcomes with various biomaterials and animal models and also the limitations of the existing systems.

## Background

*In vitro *ovarian follicle culture is a new frontier in assisted reproductive technology with tremendous potential. The ovarian cortex is populated with primordial follicles containing immature oocytes in meiotic arrest. *In vivo*, hormonal influences trigger the maturation of a single follicle to the Graafian stage and the ovulation of a single mature oocyte per cycle. During IVF treatment, hormone injections are used to stimulate the maturation of multiple follicles within the ovary [1]. Multiple mature oocytes can then be surgically extracted from the patient's ovaries. An alternate approach that is the focus of research by many fertility specialists, involves extraction of ovarian tissue/follicles and induction of the growth and maturation of oocytes *in vitro *[2]. Such technology may be especially beneficial to cancer patients, who are at risk of losing their future fertility as a result of damage to the ovary from chemo and/or radiotherapy. A potential solution for these patients is to cryopreserve intact pieces of ovarian tissue containing numerous immature follicles [3] or to cryopreserve immature follicles enzymatically isolated from this ovarian tissue [4]. Both of these techniques, however, require that the immature follicles are matured at some point and are induced to produce mature oocytes that can be fertilized. The major impediment to ovarian and follicle cryopreservation has been our limited ability to culture and eventually induce *in vitro *maturation (IVM) of the follicle/oocyte complex within the laboratory [5]. The process of IVM requires that whole follicles be grown for extended periods of time *in vitro *[6].

In the present work we review pertinent aspects of *in vitro *follicle maturation, with an emphasis on tissue-engineering solutions for maintaining the follicular unit during the culture interval. We focus primarily on presenting the various 3-dimensional (3-D) culture systems that have been applied for *in vitro *maturation of follicle:oocyte complexes. We also try to present an overview of outcomes with various biomaterials and animal models and also the limitations of the existing systems. Finally, we touch on the use of microfluidics for gamete/embryo culture and its potential application to follicular culture.

### Importance of maintaining of follicular architecture

Folliculogenesis within the ovary is a complex process requiring interaction between somatic cell components and the oocyte. At birth the human ovary contains 1-2 million primordial follicles, each containing an oocyte in meiotic arrest at the prophase stage [7]. The oocyte is surrounded by a layer of somatic granulosa cells. Follicular growth from the primordial to the pre-ovulatory stage occurs in two distinct stages. The first growth phase occurs very slowly and is not directly dependent on gonadotrophin levels [8]. There is proliferation of the granaulosa cell layer surrounding the oocyte and an increase in both follicle and oocyte diameter. This stage can take weeks in rodents and months in larger animal species, including humans. In the human, follicles increase in size from 30-50 μm in primordial resting follicles, to 100-200 μm in pre-antral follicles [9]. The second phase of follicular growth is far more rapid and culminates with the ovulation of a mature oocyte. Follicles are now responsive to follicle stimulating hormone (FSH) and luteinizing hormone (LH). The formation of a fluid filled antrum and synthesis of steroid hormones marks the transition to the antral phase of follicle development. Human follicles are over 18 mm when they reach the pre-ovulatory or Graafian stage and the oocyte is close to its final size, around 120 μM [10]. The multi-layer follicle is now surrounded by a basement membrane that separates it from the underlying vascularized thecal cell layer.

Oocyte growth and cytoplasmic meiotic competence are dependent on the gap junctions between the oocyte and the granulosa cells [11]. Knock out mice lacking the gene encoding for gap junction protein connexin-37 have impaired folliculogenesis [12]. The gap junctions connecting the granulosa cells and the oocyte enable sharing of secreted paracrine factors that promote the growth of both cell types [13-16](reviewed in [17,18]). Evidence suggests that granulosa cell proliferation and certain metabolic processes are controlled by oocyte-derived secretions [18,19]. The oocyte is unable to transport certain amino acids, carry out glycolysis and cholesterol biosynthesis without the cooperation of granulosa cells in providing necessary factors [20]. The oocyte overcomes these metabolic deficiencies by stimulating expression of specific genes in the cumulus cells that control synthesis of enzymes and amino acids that it needs. Severing of the gap junction and intercellular communication during *in vitro *culture triggers premature ovulation and eventual degeneration of the released oocyte [19].

Maintenance of the intricate 3-D architecture and granulosa-oocyte interaction may therefore be critical for successful *in vitro *maturation of follicles. In conventional 2-dimensional (2-D) tissue culture systems, the follicle tends to flatten and granulosa cells surrounding and nurturing the growing oocyte, migrate away, leaving it naked and unable to complete the maturation process [21]. This is especially true when dealing with human primordial follicles, which may need as long as three months in culture [22].

### Culture systems for follicle growth 2-D versus 3-D

The majority of early and ground breaking work on *in vitro *follicle culture was undertaken using conventional 2-D culture methodology. Pre-antral follicle growth in multi-well plates [23] as well as in microdrop culture [23-26] yields mature oocytes. Eppig and Schroeder [13] were able to achieve live births after *in vitro *maturation of mouse pre-antral follicles on a collagen impregnated gel and *in vitro *fertilization of the IVM oocytes. By including eight days of in situ culture of the intact newborn mouse ovary, the same collagen culture methodology could also be used to successfully mature primordial follicles and produce live offspring [27]. Other 2-D systems used for follicle culture include membranes coated with extracellular matrix proteins [28-31]. Despite the successes achieved with these 2D systems, they have been sub-optimal for sustained culture of cow, sheep and human follicles (reviewed in [21]). Culture on treated membranes or tissue culture substrata, impedes preservation of the spatial arrangements of cells seen *in vivo*. Follicular flattening due to granulosa cell attachment to the tissue culture vessel is problematic making the follicle complex extremely vulnerable to disruption of gap junctions. With enzymatic follicle isolation techniques, perturbation of the basal lamina surrounding the follicle can lead to granulosa cell migration away from the oocyte.

Establishing an *in vitro *culture model that can more accurately mimic the *in vivo *ovarian growth environment has therefore been the focus of much research. To this end, a tissue bioengineering approach has attracted much interest. The recognition of the importance of spatial arrangements between cells has spurred research in to 3-D culture systems. Data from a variety of different cellular models indicate that 3-D culture modulates cell behavior, growth, secretions, response to stimuli and communication with surrounding cells. In a landmark study, investigators were able to block the cell surface receptor β-1 integrin and completely alter the behavior of breast cancer cells grown in 3-D culture, in a manner never observed during conventional 2-D culture [32,33]. Others have noted that the gene expression profile of cells grown in 3-D culture more closely resembles that seen *in vivo *[34] and distinctly differs from that found after conventional 2-D culture. In addition to the spatial arrangement of the cells it is becoming increasingly evident that the extracellular matrix support structure (ECM) plays a defining role in organizing communication between cells, controlling cell differentiation and modulating response to biochemical signals from the cellular microenvironment (reviewed in [35]).

**Figure 1:**
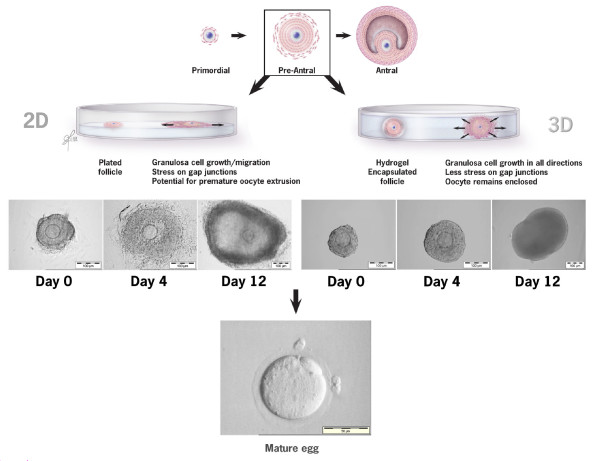
**A comparison of follicular growth in a 2-D vs 3-D culture system**. With 2-D growth granulosa cell migration away from the oocyte is evident with time in culture, leaving the oocyte vulnerable for premature extrusion. Pre-antral follicles embedded in hydrogel maintain their 3-D architecture. Granulosa cell expansion occurs in all directions resulting in less stress on gap junctions.

Scaffolding, matrix proteins and 3-D culture systems to maintain follicular architecture are avenues of research currently being explored by numerous investigators to gain further insight on the growth requirements of follicles. These 3-D systems are characterized by their ability to maintain the spherical morphology of the ovarian follicle and preserve the critical cell-cell and cell-matrix interactions within the surrounding stromal tissue, thereby allowing follicles to successfully complete the maturation process [21]. Figure 1 depicts growth of pre-antral follicles in a 2-D vs 3-D culture system. Encapsulation of follicles may protect them from gap junction disruption through shear stress [36,37] and may preserve expression of the gene encoding for the gap-junction protein connexin [38]. Contiguous assembly of granulosa cells around the oocyte also prevents the follicles from undergoing premature ovulation [39].

Another advantage may be that trophic factors released by granulosa cells remain in close proximity to the oocyte exerting a positive effect on oogenesis and possibly fostering new local gap junctions. This is an advantage not shared by 2-D culture vessels where the volume of medium in culture vessels and polarization of granulosa cells towards the culture substrata may result in a more diffuse and less uniform exposure to secreted factors.

Despite the potential advantages with 3-D culture, there is still a good deal of controversy as to how best to achieve such a culture system. The questions revolve around the type of biomaterials available, their characteristics, permeability, toxicity and ability to be molded and handled with ease during follicle loading and harvest. In addition, their biologically usefulness ultimately depends on survival of the follicle and maturation of the oocyte *in vitro*. The animal species and the length of time needed for follicular culture also warrant consideration in determining whether 3-D culture will be beneficial. In humans, *in vitro *follicle maturation from primary to the antral stage can take more than 120 days [22], in contrast to 30 or so days for follicle maturation in rodent species (reviewed in [40]). Moreover, by the early antral stage human follicles measure 2-5 mm in diameter. Active perfusion systems may therefore prove necessary to assure sufficient nutrient supply to multi-layered follicles if cultured in a 3-D environment.

### Design parameters for biomaterials for 3-D culture

Chemical and physical properties of biomaterials present certain design limitations that must be meshed with the physiologic needs of the follicular unit. First and foremost, the chemical composition must be non-cytotoxic, allowing sustained cell viability for extended culture periods. Growth of follicular diameter during the course of *in vitro *maturation dictates materials with a certain amount of elasticity allowing expansion of the granulosa cell layers, yet providing enough support to retain spherical shape and prevent inadvertent denudation of the oocyte. This is especially important in 3-D culture systems that physically encapsulate the follicle within a biomaterial. In addition to maintaining structural integrity, this biomaterial must allow adequate gas exchange, diffusion of nutrients and removal of cellular waste. Within the ovary there is an increase in vascularization as one moves deeper in to the ovarian cortex where secondary and pre-antral follicles grow [41]. This suggests a stronger need for oxygen diffusion during the final stages of follicle maturation [37]. The need for oxygenation may also require an active perfusion system when dealing with longer *in vitro *maturation intervals.

Diffusion across the biomaterial during 3-D culture is controlled by creating specific pore sizes [42]. The mechanical properties of the biomaterial, such as viscosity and its ability to be molded also contribute to its usefulness for follicle culture and are dependent on molecular weight [43].

Another important attribute is the biomaterial's rigidity, also referred to as shear modulus. The shear modulus of a biomaterial is a mathematical description of its elastic properties-that is its ability to resist deformation with the application of a force. Biomaterial rigidity and its effect on follicle diameter, theca formation, antrum formation, estradiol production, and rate of meiotic resumption (GVBD and metaphase II oocyte formation) can all be used to compare outcomes with follicle encapsulation in 3-D culture models [44-46].

The ideal biomaterial for *in vitro *follicle maturation would also be one that could mimic the extracellular matrix (ECM) found within the ovary [47]. It has been suggested that since ovarian stromal composition can vary, the selected ECM for follicle culture should ideally share the inherent properties of the particular species being cultured [48].

A final consideration is whether to individually or co-culture follicles. Culturing follicles in clusters allows sharing of autocrine/paracrine secretions, increasing follicle-to-follicle communication and possibly enhancing the culture environment [49]. However, some disadvantages of co-culture systems include the potential sharing of growth-inhibiting hormones, like AMH (anti-Mullerian hormone), amongst follicles [50]. Also co-culture can interfere with the monitoring, tracking and harvesting of individual follicles during the maturation process.

## Current 3-D culture models

### In situ culture

*In vitro *culture of ovarian tissue pieces as a technique for in situ 3-D follicle maturation has not been very effective. While primordial follicle growth can be supported in this manner, development of follicles past the pre-antral stage is inhibited. To obtain complete *in vitro *maturation of follicles and the release of a metaphase II oocyte it is necessary to remove the follicle from the ovarian cortex [51,52]. Two-step culture systems in which follicles are first grown from the primordial stage in situ and then mechanically or enzymatically isolated and grown *in vitro *have been investigated with mouse as well as human ovarian tissue [49,51]. Models for human *in vitro *follicle maturation from primordial or even pre-antral stages to a mature oocyte are still in the early phase. Recently, Li and colleagues were able to induce maturation of primordial follicles in ovarian tissue fragments from cancer patients. Human ovarian tissue fragments were treated with PTEN gene inhibitor and transplanted to immunodeficient mice [53]. The PTEN (phosphatase and tensin homolog) gene plays a pivotal role in cell regulation and apoptosis. Interestingly, in this study, inhibition of the PTEN gene allowed primordial follicles to advance to the pre-ovulatory stage. The ability to activate dormant ovarian follicles may play a pivotal role in establishing a successful *in vitro *culture model for maturing primordial follicles. A much deeper understanding of factors regulating human folliculogenesis is still needed to successfully mature human follicles to the Graafian stage and to be able to assess fertilization potential. Progress in this arena may well depend on establishing adequate 3-D culture systems that preserve the normal follicular architecture and allow extended *in vitro *culture intervals.

### Matrices for follicle culture

Synthetic and biologic matrices for the support of follicle growth and maturation have been studied in several animal models as well as in humans. Table 1 presents various 3-D systems and matrices that have been applied to the culture of rodent pre-antral follicles. Table 2 summarizes 3-D culture models for follicles from larger animals, primates and humans.

**Table 1:** Studies examining matrices for 3-D culture of pre-antral ovarian follicles from rodents

Author	Year	System Description	Species	Culture Period	Initial Follicle Diameter	Survival rate (%)	GVBD (%)	MII Formation (%)	Additional Outcomes/ Observations
Jin et al. [102]	2010	In situ culture followed by encapsulation of follicles in 0.25% ALG-alginate or FA-fibrin alginate	Mouse	In situ: 4 days Alginate: 12 days	NA	Alginate: 69.6% FA: 75%	Alginate: 75% FA: 86% (from follicles with antrum formation)	Alginate: 61.3 ± 2.4 FA: 88 ± 8.7 (from oocytes undergoing GVBD)	Formation of 2 cell embryos Alginate: 33% FA: 54 ± %
Xu et al. [73]	2009	Fresh follicles (Foll) and cryopreserved (Cryo-Foll) and crypreserved ovarian tissue (Cryo-OV) ALG-alginate (0.25%)	Mouse	12 days	100-130 μm	Fresh-Foll 78% Cryo-Foll 74% Cryo-OV 72%	Fresh-Foll: 83% Cryo-Foll 69% Cryo-OV: 92%	Fresh-Foll:59% Cryo-Foll: 64% Cryo-Ov 68%	Gap junction protein, connexin expression also studied-down regulated after cryopreservation
Shikanov et al. [103]	2009	ALG (0.25%) FA fibrin alginate	Mouse	12 days	100-130 μm	ALG:78% FA:77-81%	ALG:88% FA: 72-88%	ALG:67% FA:75-82%	NA
West et al. [45]	2007	ALG (0.7%, 1.5%, 3%)	Mouse	8-12 days	100-130 μm 150-180 μm	100-130 μm 15-42% 150-180 um 83-91%	100-130 μm 31-66% 150-180 um 46-91%	NA	Low matrix stiffness increase growth, antrum, GVBD and E2
Kreeger et al. [75]	2006	Tested alginate with ECM 2 versus multi-layer follicles ALG alginate alone (1.5%) CI collagen I FN fibronectin RGD peptides CIV collagen IV LN laminin	Mouse	8 days	100-130 μm versus 150-180 μm	ALG 64% vs 69% CI 65% vs 67% FN 70% vs 72% RGD 72% vs 62% CIV 72% vs 48% LN 63% vs 61%	ALG 12% vs 38% CI 18% vs 25% FN 23% vs 17% RGD 13% vs 36% CIV 20% vs 36% LN 29% vs 11%	ALG 40% CI 44% FN 71% RGD 65% CIV 50% LN 71% *Multi-layer follicles	Transition to secondary Follicle promoted by CI and RGD MII formation promoted by: FN, RGD, LN
Xu et al. [39]	2006	ALG (1.5%)	Mouse	8 days	150-180 μm	93%	82%	71%	Live births of pups
Xu et al. [46]	2006	ALG (0.25%, 0.5%,1%.1.5%)	Mouse	12 days	100-130 μm	74-85%	78-88%	56-67%	Decreasing 2-cell and blast with increasing % ALG
Heise et al. [36]	2005	ALG (1%) Encapsulation with or without FSH in gel medium	Rat	72 hrs	150-160 um	NA	NA	NA	Inclusion FSH with ALG and culture medium, Follicle diameter increased by 33%
Mousset-Simeon et al. [26]	2005	Microdrops under oil Agar Millicell-CM membrane insert	Mouse	12 days	100-130 μm	Microdrops 72% Membrane 46% Agar 30%	Microdrops 63% Membrane 33% Agar 26%	Microdrops 53%, Membrane 56% Agar 13%	2-D Microdrop high survival. Maturation rate similar to 3-D on membrane
Kreeger et al. [71]	2005	Compared effects two versus multi-layered follicles ALG(1.5%)-Collagen I matrix FSH 5-50 mIU/ml	Mouse	8 days	100-130 μm 150-180 μm	66-77% 30-72%	21-27% 9-43%	Multi-layer follicles 40-78%	Hormone secretion (E2 and progesterone) in multi-layer follicles FSH dependent
Loret de Mola et al. [63]	2004	Collagen treated membrane Collagen gel encapsulation	Mouse	10 days	118 μm	Membrane 55% Collagen gel 15%	NA	Membrane 17% Collagen gel 19% *Based on number of recovered eggs	Follicle size larger in collagen gel but maturation and rate of 2-cell formation not enhanced
Adam et al. [25]	2004	Microdrops under oil Millicell-CM membrane insert	Mouse	6 days	150-174 μm 175-200 μm	Microdrops 77% Membrane 83%	NA	Membrane 79%	Membrane insert Fert rate 75%, blast rate 48%
Pangas et al. [70]	2003	Alginate	Mouse	10 days	82 μm	68%	NA	40%	TEM indicate follicles in ALG maintained ultrastructure
Gomes et al. [62]	1999	Collagen gel encapsulation	Mouse	6 days	135 μm	NA	NA	NA	Follicle volume and response to FSH increased with 3-D culture in collagen
Nayudu et al. [54]	1992	Millicell-CM membrane insert	Mouse	6-7 days 3-5 days	125-150 μm 150-180 μm	NA	NA	NA	FSH stimulated growth, antrum formation, E2 dose response to FSH levels
Torrance et al. [61]	1989	Collagen gel encapsulation	Mouse	14 days	20-95 μm	36%	NA	NA	Growth to multi-laminar stage but no antrum formation

NA: Not available

**Table 2:** Summary of 3-D culture studies with follicles from human, primate and large domestic animal species

Author	Year	System Description	Species	Culture Period	Initial Follicle Diameter	Final Diameter	Survival Rate	Antrum Formation	Observations/Conclusions
Amorim et al. [72]	2009	Alginate (ALG)1%	Human	7 days	34-52 μm	44--70 μm	90%	NA	Alginate culture system supported growth of Isolated follicles from frozen-thawed ovary
Xu et al. [67]	2009	Alginate 0.5% Matrigel embedded	Human	30 days	~175 μm	715 μm	NA	75%	Both 3-D systems supported growth of isolated human follicles
Xu et al. [48]	2009	Alginate (ALG) 0.25% versus 0.5%	Rhesus monkey	30 days	100-300 μm	20 vs 78%	60 vs 78%	Yes	Higher ALG better survival and growth. LH addition with FSH negative effect on survival and P_4 _secretion
Itoh et al. [104]	2002	Collagen gel	Cow	13 days	145-170 μm	304 μm	NA	Yes	Serum-free culture. Insulin, FSH and LH together induced earlier antrum formation
Abir et al. [51]	2001	Collagen gel	Human	24 hours	35-45 μm	70 μm	NA	NA	Collagen matrix supported growth of fully isolated follicles but not tissue slice with partially isolated follicles
Hovatta et al. [49]	1999	In situ and partially isolated follicles Millicell + Matrigel	Human	~28 days total	NA	NA	NA	No	Tissue slices better less oocyte extrusion than collagenese isolated. Four weeks to reach secondary stage
Yamamoto et al. [105]	1999	Collagen gel	Cow	14 days	500-700 μm	NA	37%	Yes	MII 27%, 42% fertilization, 4% blastocyst One live birth.
Hiraoi et al. [106]	1994	Collagen gel	Pig	16 days	220-300 μm	NA	NA	Yes	40% MII formation in oocytes ≥110 μm No MII from oocytes <110 μm. Oocytes capable of being fertilized
Roy and Treacy [107]	1993	Agar	Human	5 days	90-220 μm	NA	NA	Yes	FSH induced antrum formation, hormone secretion. No FSH, no E2 secretion

All of the matrices adopted for 3-D culture essentially permit spherical growth of the follicle, preserving the physical integrity of granulosa cell and oocyte's interaction. Nayadu et al [54] accomplished this using a Millicell hydrophobic insert. The non-tissue culture treated surface prevented granulosa cell migration that could disrupt follicle architecture.

A variety of optically clear gels have also been applied towards follicle culture in different animal models. Follicles have either been completely encapsulated to create a 3-D environment or grown on a gel membrane with medium bathing both surfaces to simulate 3-D culture. Gels that have been used for tissue engineering include hydrogels like agar/agarose, calcium alginate, and hyaluronan, all from naturally derived polymers, as well as synthetic polymers such as poly (ethylene glycol) and poly (vinyl alcohol) (reviewed in [21,55]). Gels containing collagen alone as well as compounds containing collagen in combination with ECM proteins have also been applied to *in vitro *follicle growth. The physical characteristics of each of these matrices permit physical expansion of the follicular unit during growth.

Hydrogels contain polymers that cross-link or self-assemble into hydrophilic structures. The 3-D cross-linking is what gives the gel its stiffness. The temperature and conditions for this cross-linking can be a critical factor in determining subsequent development of the follicle. For instance agar, derived from seaweed, requires exposure to elevated non-physiologic temperatures for melting before the cross-linking or gelling step, potentially damaging the follicle [56]. Higher rates of atresia were observed in follicles grown on agar as compared to those placed in microdrop culture or in 3-D culture on a hydrophobic membrane insert [26]. In contrast, Huanmin et al. (2000) described active follicular growth and antrum formation with caprine follicles embedded within agar [57]. Their data did however show that secondary follicles survived better than primary follicles in this 3-D agar culture system. Agar embedding has also been applied to human and hamster pre-antral follicles [58,59]. Follicles were biologically competent, secreting steroids and synthesizing DNA. Low melting point agarose may be a better matrix for follicle embedding, permitting encapsulation at temperatures more conducive to continued cell growth.

Collagen, a protein found in fibers of connective tissue, is rich in glycine and proline and can be hydrolyzed in to a gel by boiling. This biomaterial has been widely applied to follicular culture. Eppig and colleagues used collagen membrane inserts as substrata in an attempt to simulate 3-D follicle culture. The membrane inserts with follicles were suspended in wells, and follicles were exposed to culture medium from below as well as above [13,60]. The biomaterial was not tissue culture treated. It did however allow follicle attachment but minimized granulosa cell migration. *In vitro *follicle maturation resulted in the formation of metaphase II oocytes, with the capability of producing live young after *in vitro *fertilization, growth and transfer to foster mothers. Despite this achievement, follicle growth on collagen treated membranes had limited potential in terms of maintaining spheroid follicle structure and follicles were susceptible to flattening over time in culture and to premature oocyte ovulation.

To create a more spatially uniform 3-D culture system, follicles have also been embedded in collagen gel [61-64]. Spontaneous follicle disruption as a result of discontinuous or distorted basal lamina and granulosa cell migration was decreased in the 3-D collagen system compared to control 2-D culture systems [62]. Follicle growth rate has also been reported to be superior [63]. Granulosa-cell oocyte complexes embedded in collagen matrix remained rounded and compacted with neuronal-like outgrowths towards the oocytes [64]. Two limitations of the collagen gel have however been noted. The collagen gel is susceptible to shrinkage over time, affecting the gel's natural properties as well as reducing visibility during microscopic assessment [61]. Also, follicle extraction from the collagen requires enzymatic digestion of the gel, with the potential for subsequent damage to the oocyte [65].

The natural scaffolding upon which cells are organized *in vivo*, known as the extracellular matrix (ECM), is composed of collagen, along with laminin and fibronectin. ECM has been shown to play an important role in regulating cell behavior, differentiation and secretory activity (reviewed in [30]). One commercially available ECM tested for follicle growth is matrigel [49,66,67]. This ECM product is derived from the Engelbreth-Holm-Swarm (EHS) mouse sarcoma. Matrigel is composed of collagen IV, laminin, fibronectin, entactin, heparin sulfate proteoglycans, and a variety of growth factors such as EGF, FGF, IGF-1, PDGF and TGF-β [68,69]. Murine pre-antral follicles in 3-D culture in matrigel exhibited higher growth and survival rates than those in conventional culture [28]. Hovatta et al demonstrated higher survival of follicles in frozen-thawed human ovarian tissue placed in culture on matrigel coated inserts [29,49]. Autocrine and paracrine signaling by ECM molecules and associated growth factors likely affect folliculogenesis. The interactions between ECM proteins and follicles from different animal models needs to be further studied. The source and type of ECM could also play a role in regulating follicle growth during 3-D culture. The size of ECM molecules can present problems and an alternative solution has been to adsorb known sequences of matrix peptides, such as RGD (Arg-Gly-Asp) or laminin-derived peptide sequences on to synthetic matrices (reviewed in [30]).

To date the most widely applied system for follicle encapsulation and 3-D culture has been alginate produced by brown algae [39,45,46,48,67,70-73]. Alginate in the presence of calcium crosslinks to form a hydrogel. This property facilitates encapsulation of follicles under physiologic conditions. Pangas et al. (2003) first applied this system to the 3-D culture of granulosa-cell oocyte complexes (GOC) from 12-day old mouse pre-antral follicles [70]. GOCs were embedded in alginate beads ranging in size from 0.5 to 1 mm in diameter. Light microscopic and TEM ultra-structure studies suggested that the alginate did not interfere with oocyte or granulosa cell growth development over a 10 day culture interval. Moreover, oocytes recovered from the encapsulated GOCs were able to resume meiosis, undergo fertilization and produce viable offspring [39]. This 3-D system has also been applied to secondary follicles. Follicles embedded in alginate hydrogels responded to FSH stimulation in a dose-dependent fashion, secreting estradiol and progesterone [71]. Alginate matrix stiffness and density can affect secondary follicle expansion, hormone production and oocyte maturation [45,46]. Non-human primate follicles have also been successfully cultured in calcium alginate gels for up to 30 days [48]. The encapsulated monkey pre-antral follicles secreted estrogen, progesterone and androstenedione and responded to FSH in the culture milieu. Interestingly, follicles cultured in 0.5% alginate performed better than those in 0.25% alginate, suggesting that primate follicles may require more physical support. One concern however is that denser matrices could potentially limit access to hormones and other nutrients. Heise et al. (2005) reported inhibited delivery of FSH to micro-encapsulated follicles [36]. Follicle diameters increased with inclusion of FSH in the hydrogel but still did not reach that observed in un-encapsulated controls. Clearly, the physical attributes of the 3-D matrix selected for follicle culture needs to be tailored towards the species and follicle stage being cultured.

In humans, pre-antral follicle growth *in vitro *offers an avenue through which cryopreserved ovarian tissue can be utilized without the need for transplantation. Human follicles isolated from fresh or cryopreserved ovarian tissue have been successfully cultivated in calcium alginate hydrogels but functionality needs to be further characterized [72]. Initial data with frozen mouse ovarian tissue certainly suggests that meiotically competent oocytes can be recovered after *in vitro *maturation of isolated follicles in this 3-D culture system [73].

To further simulate the *in vivo *environment, ECM molecules have been combined with calcium alginate to construct synthetic ECM matrices for 3D culture [74]. The adhesion peptide sequence arginine-glycine-aspartic acid (RGD) common to ECM proteins has been synthetically created and coupled to calcium alginate to construct such a synthetic matrix for follicle growth. Hormone secretion by follicles was directly related to adhesion peptide concentration and a three-fold increase in progesterone and estradiol secretion could be induced by adjusting matrix parameters. In a separate study, these investigators combined calcium alginate with additional ECM components such as collagen I, collagen IV, laminin and fibronectin [75]. Matrix effect on growth from two-layered to multi-layered follicles as well as oocyte maturation to metaphase II was compared. Transition to the multi-layered, secondary follicle was enhanced in alginate matrices with RGD or collagen I. Final maturation of oocytes and resumption of meiosis was promoted by presence of fibronectin, laminin or RGD peptide.

### Criteria for biomaterial evaluation

Increasing follicular diameter is typically used as a measure of follicle maturation. During *in vitro *growth, especially in traditional 2-D culture systems where there is granulosa cell expansion, an increase in horizontal diameter of the follicle does not necessarily correlate to overall follicular growth [62]. With 3-D culture the biomaterial presents equal counter-forces in all directions, minimizing flattening and allowing equal growth along all axes. Follicle volume as well as diameter should therefore be taken into account when comparing different substrata.

Another outcome measure indicative of follicle functionality and growth is antrum formation. This accumulation of fluid within the follicle complex has been shown to vary with 2- versus 3-D culture systems, as well as the biomaterial used for follicle encapsulation. The shear elastic modulus and diffusion characteristics of the biomaterial must be carefully balanced. Torrance et al. (1989) noted no antrum formation in follicles cultured in collagen, despite an apparent increase in follicular diameter over the 14 day culture interval. It was suggested that the double gelling of the collagen during follicle encapsulation allowed just enough flexibility for some granulosa cell proliferation, but that the overall high shear elastic modulus (increased stiffness) inhibited antrum formation [61]. Interestingly, this was not observed when follicles were individually cultured in collagen microbeads [76].

A relationship between decreased gel stiffness and greater antrum formation was also observed with calcium alginate hydrogel when tested at concentrations of 3%, 1.5% and 0.7% [45]. The study of Xu et al. (2006) most clearly illustrates the opposing influences of the rigidity of the biomaterial at high gel concentration and its interference with diffusion and optimal growth [46]. Oocytes obtained from follicles encapsulated in 0.25% alginate had a higher developmental capacity than those cultured in 1.5% alginate. *In vitro *maturation and fertilization of oocytes in 0.25% vs 1.5% calcium alginate were significantly higher (41% vs 5%, respectively). Moreover oocytes derived from the stiffer gel were clearly impaired and unable to undergo *in vitro *blastulation [46].

Interestingly, follicles from primates showed the opposite relationship between gel rigidity and follicle growth. Follicle survival and diameter were increased with culture in 0.5% calcium alginate as compared to 0.25% [48]. Ovarian stroma of primates is more rigid than that found in rodents and it has been suggested that perhaps primate as well as human follicles may require a stiffer biomaterial to optimize *in vitro *culture and growth. The 100% survival rate and 75% antrum formation observed with human secondary follicles grown in 3-D culture in 0.5% calcium alginate matrix further support this supposition [67].

### Non-gel culture systems

Despite the aforementioned benefits of follicle encapsulation as a model for 3-D culture, there are also difficulties. The process of encapsulation as well as the removal of follicles from the gel can be problematic, sometimes resulting in loss of healthy follicles [61,63,65].

Alternatives methods for 3-D culture of follicles that do not involve encapsulation have therefore also been explored. Suspension culture of follicles in orbiting test tubes [36,37,77], rotating-wall vessels [77], and roller bottle systems [23] can maintain the 3-D morphology of the follicles without encapsulation. Unfortunately these systems have not been extremely effective. The rate of rotation necessary to keep the follicles from descending to the bottom of the vessels imposes shear stress on the follicles causing follicle degeneration [77]. Moreover, the only way to negate this effect was to encapsulate the follicles before subjecting them to suspension culture with rotation [37,77].

Suspension culture in rotating systems with its accompanying shear stress resulted in more follicle loss than that observed with embedding and removal of follicles from gels. Follicle survival with culture in a rotating-wall culture vessel was only 9% [77] as compared to the 15% observed after embedding and removal from collagen gel culture [63]. With marsupial follicles, survival rate in the roller culture system was higher; nearly 49%, but follicles exhibited no antrum formation [23].

Other non-gel approaches have included serial culture of follicles in new wells each day to prevent attachment [78] and flattening, or culture in simple microdrop under an oil overlay [79]. Inverted microdrop suspension culture has also been tested as a means to maintain the 3-D architecture of follicles [23,80]. Follicles are placed in microdrops under oil on the bottom of a tissue culture plate and then hung upside down during culture. Oil is ideally suited as a biomaterial for micro-culture environments, allowing maintenance of pH and temperature around the follicle and free gas exchange [81]. However, its hydrophobic properties could potentially allow the escape of lipid soluble follicle secretions and growth factors in to the oil layer, ultimately hindering growth [82]. It should however be noted that while inverted suspension culture yielded survival rates similar to that observed with alginate gels, the meiotic maturation rate was only 10% [23], far less than that what has been achieved with gel encapsulation of follicles [26,39,70]. Handling large numbers of follicles in inverted suspension culture would also be a delicate and labor intensive process. This method would be especially unsuitable for follicles from the human ovary, which might require as long as three months of culture.

### Microfluidic culture

The final aspect of follicle culture that needs some attention is the development of culture vessels or systems that maximize diffusion of nutrients and gases through the selected biomaterial while allowing retention of the delicate micro-environment of the follicle and the concentration of essential trophic factors around the oocyte. To accurately mimic the *in vivo *ovarian environment, fluid flow across the encapsulated follicle is vital. Also, within the ovarian environment follicles are grown in close proximity of each other, allowing sharing and concentration of secreted factors. The logistics of co-culturing numerous encapsulated follicles can perhaps be aided by the use of microfluidics that allow precise control and manipulation of fluids using microchannels. Microchannels increase the surface area-to-volume (SAV) ratio, implementing laminar fluid flow [83](reviewed in [84]).

Diffusion across biomaterials has been shown to be influenced by not only the biomaterial and its concentration but also by its shape or presentation. Encapsulating in microbeads of gel may allow more uniform diffusion across all surfaces as compared to culture with follicles embedded in a single continuous layer of gel. Survival and antrum formation by cultured pre-antral buffalo follicles was demonstrated to be better after culture in collagen microbeads as compared to a continuous layer of collagen matrix [76]. Tiny microbeads containing follicles in a biomatrix, combined with a system of microchannels could be used to create a network of individual follicles sharing nutrients. A dynamic medium exchange could therefore be applied to follicle culture in a manner that avoids the shear stress observed with rotating culture systems and preserves a "co-culture" atmosphere.

A variety of microfluidic culture systems have been described. Cell immobilization with continuous media flow is the common goal. This can be accomplished with microposts on the culture surface to entrap cells and create a matrix support while still allowing laminar flow of fluid to pass by [85,86] or by entrapping cells between walls of PDMS with continuous flow of culture medium above the cells [83]. Microwells can also be used as architectural supports in microfluidic systems and act as nests for cells to culture in while fluid is exchanged above or below [87,88]. Microfluidics in combination with valves and micro-scale pumps provide the option of continuous media flow in ways similar to that seen *in vivo *[89,90]. Microfluidics thus permits dynamic culture conditions and medium flow without disturbing the cell itself.

Application of microfluidics to the field of reproductive biology has gained much attention. It has been applied to sperm sorting [91,92], oocyte handling and fertilization [93-96] and embryo culture [83,97-101]. Follicle culture in microfluidic devices needs to be explored. This type of system may be ideal for providing the 3-D environment necessary for maintaining follicle architecture over long intervals in culture, allowing adequate oxygenation and nutrient exchange and at the same time permitting sequestration of autocrine/paracrine factors within the vicinity of the growing follicle. The ideal microfluidic model would allow monitoring and harvest of individual follicle but also a sharing of the microenvironment to attain the benefit of "co-culture".

## Conclusion

In conclusion, a review of the literature suggests that 3-D culture of encapsulated follicles offers much promise. Further study and selection of the appropriate biomaterial with the chemical and physical properties necessary for follicle handling and growth will be a key to making advances in this field. The combination of synthetic ECM matrices with a microfluidics model may be necessary to further simulate the *in vivo *environment and improve *in vitro *follicle maturation, especially in the human.

## Competing interests

The authors declare that they have no competing interests.

## Authors' contributions

ND critically reviewed publications. AA summarized articles. FA critically reviewed publications. AC critically reviewed manuscript. JG critically reviewed manuscript. AF critically reviewed manuscript. TF critically reviewed manuscript. All authors read and approved the final manuscript.
